# Octulose: a forgotten metabolite?

**DOI:** 10.1093/jxb/erx367

**Published:** 2017-11-11

**Authors:** Qingwei Zhang, Dorothea Bartels

**Affiliations:** Institute of Molecular Physiology and Biotechnology of Plants (IMBIO), University of Bonn, Germany

**Keywords:** Carbohydrates, octulose synthesis, pentose phosphate pathway, resurrection plants, sugar metabolism, transaldolase, transketolase

**The monosaccharide octulose was first isolated and identified about seventy years ago. Although this sugar has frequently been found in plants, bacteria, yeast and animals, its metabolic function has hardly been explored. It often occurs in small amounts, but occasionally it is highly abundant, notably in some resurrection plants. Recent results show that its synthesis may involve an alternative pentose phosphate pathway and that when present in large amounts it might confer important benefits such as ROS scavenging. It could also have value for nutrition and healthcare. The time is ripe for developing our understanding further.**

The eight-carbon monosaccharide octulose was found in plants, animals and humans many years ago (Bartlett and Bucolo, 1960; Charlson and Richtmyer, 1960; Bartlett, 1970). As an eight-carbon ketose, it can exist in isomeric forms; except for the 3-octulose D-*gluco*-L-*glycero*-3-octulose found in young leaves of *Laurus nobilis* (Sakata *et al.*, 1989), the other reported octulose isomers in plants are all 2-octuloses and include D-*glycero*-D-*manno*-octulose, D-*glycero*-L-*galacto*-octulose, L-*glycero*-L-*galacto*-octulose, D-*glycero*-D-*altro*-octulose and D-*glycero*-D-*ido*-octulose (Table 1; see structures in Fig. 1). The amount present varies among plant species – for instance, only about 1 g of D-*glycero*-D-*manno*-octulose was obtained from 27 kg of avocado fruit (Charlson and Richtmyer, 1960), while D-*glycero*-D-*ido*-octulose makes up about 90% of total sugars in hydrated leaves of the resurrection plant *Craterostigma plantagineum* (430 mg g^–1^ lyophilized leaf material) (Bianchi *et al.*, 1991).

**Table 1.** Octulose-containing plant species

Species	Octulose isomers	Source for octulose extraction	References
*Craterostigma plantagineum*, *C. agnewi* and *C. pumilum*	D-*glycero*-D-*ido*-octulose	Hydrated and dried leaves	Bianchi *et al.*, 1991; Egert *et al.*, 2015
*Fabiana imbricata*	D-*glycero*-D-*manno*-octulose	Dried herbage	Richtmyer, 1970
*Laurus nobilis*	D-*gluco*-L-*glycero*-3-octulose	Flush (young leaves) and leaves	Sakata *et al.*, 1989
*Lindernia brevidens*, *L. numilarifolia*, *L. philcoxii* and *L. subracemosa*	D-*glycero*-D-*ido*-octulose	Hydrated and dried leaves	Kutzer, 2004; Phillips *et al.*, 2008
*Lindernia acicularis* and *L. exilis*	D-*glycero*-D-*ido*-octulose	Dried leaves	Kutzer, 2004
*Medicago sativa*	D-*glycero*-D-*manno*-octulose	Leaf petiole fraction of young shoots	Rendig *et al.*, 1964
*Papaver somniferum*	D-*glycero*-D-*manno*-octulose	Capsules	Haustveit and Wold, 1970
*Persea americana* (avocado)	D-*glycero*-D-*manno*-octulose, D-*glycero*-L-*galacto*-octulose	Fruits	Charlson and Richtmyer, 1960; Sephton and Richtmyer, 1963*a*
*Phalaris tuberosa*	D-*glycero*-D-*manno*-octulose	Dry leaves	McComb and Rendig, 1970
*Primula officinalis*	D-*glycero*-D-*manno*-octulose, D-*glycero*-L-*galacto*-octulose	Dried roots	Begbie and Richtmyer, 1966
*Sedum spectabile*	D-*glycero*-D-*manno*-octulose, D-*glycero*-L-*galacto*-octulose	Whole plants	Charlson and Richtmyer, 1960; Sephton and Richtmyer, 1963*b*
*Spinacia oleracea*	D-*glycero*-D-*altro*-octulose, D-*glycero*-D-*ido*-octulose	Leaves and isolated chloroplasts	Arora *et al.*, 1985; Flanigan *et al.*, 2006; Williams and MacLeod, 2006
*Trifolium pratense*	D-*glycero*-L-*galacto*-octulose	Leaves supplied with D-gulose and D-xylose	Haustveit *et al.*, 1975*a*
*Trifolium pratense*	L-*glycero*-L-*galacto*-octulose	Leaves supplied with L-mannose and L-arabinose	Haustveit *et al.*, 1975*a*
*Trifolium pratense*	D-*glycero*-D-*altro*-octulose	Leaves supplied with D-ribose and D-allose	Haustveit *et al.*, 1975*b*

**Fig. 1.**
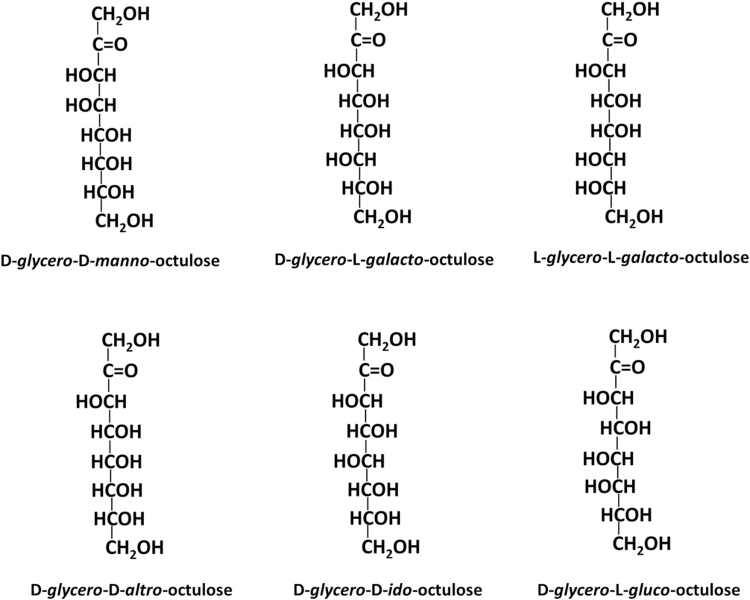
Structures of octulose isomers described in this article (Jones and Sephton, 1960; Flanigan *et al.*, 2006).

For the reported octulose-containing plant species, their relatives in the same genus or family may also contain the sugar. For example, except *Lindernia rotundifolia*, six investigated *Lindernia* species have been shown to synthesize D-*glycero*-D-*ido*-octulose (Table1: Kutzer, 2004; Phillips *et al.*, 2008). Similarly, in the genus *Craterostigma*, three species synthesize substantial amounts of octulose in the hydrated state (Table 1: Egert *et al.*, 2015). It is certainly reasonable to assume that it occurs in more higher plants than known to date.

## Metabolic pathways involving octulose

**Fig. 2.**
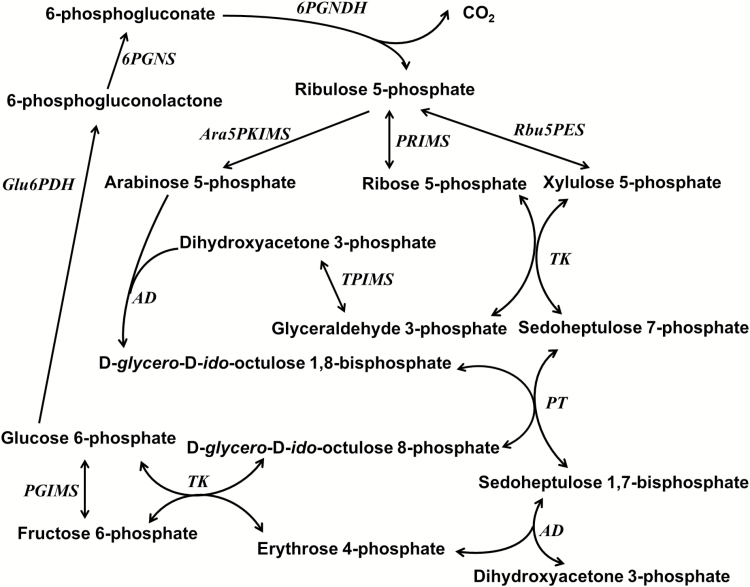
The L-type pentose phosphate pathway based on the report by Williams and MacLeod (2006). 6PGNDH, 6-phosphogluconate dehydrogenase; 6PGNS, 6-phosphogluconolactonase; AD, aldolase; Ara5PKIMS, arabinose 5-phosphate ketolisomerase; Glu6PDH, glucose 6-phosphate dehydrogenase; PGIMS, phosphoglucose isomerase; PRIMS, phosphoribose isomerase; PT, phosphotransferase; Rbu5PES, ribulose 5-phosphate 3-epimerase; TPIMS, triose phosphate isomerase; TK, transketolase.

When octulose was first found to be present in various plant species, successful attempts were made to synthesize it *in vitro* (see Box 1). The possible physiological role of the sugar has mainly been explored by isotope labeling and GC/MS. Williams *et al.* (1978) proposed an alternative to the pentose phosphate pathway: this L-type pentose phosphate pathway in rat liver includes arabinose 5-phosphate, sedoheptulose 1,7-bisphosphate and the mono- and bisphosphates of D-*glycero*-D-*ido*-octulose as intermediates (Fig. 2). Williams and MacLeod (2006) proposed that D-*glycero*-D-*ido*-octulose 8-phosphate, D-*glycero*-D-*altro*-octulose 8-phosphate and D-*glycero*-D-*altro*-octulose 1,8-bisphosphate may also be reactants in a modified Calvin–Benson–Bassham pathway in spinach in which D-*glycero*-D-*ido*-octulose 8-phosphate is synthesized by the exchange reaction catalysed by transketolase using fructose 6-phosphate and glucose 6-phosphate as substrates, and D-*glycero*-D-*altro*-octulose 1,8-bisphosphate is synthesized via aldolase with ribose 5-phosphate and dihydroxyacetone phosphate as substrates (Flanigan *et al.*, 2006). This reaction was confirmed using the recombinant *C. plantagineum* transketolase 7 and 10, which catalyses the exchange reaction and produces D-*glycero*-D-*ido*-octulose 8-phosphate using glucose 6-phosphate and fructose 6-phosphate as substrates (Zhang *et al.*, 2016). Octulose phosphate was also identified in the protozoan parasite *Trypanosoma brucei*, and it was shown that octulose 8-phosphate is synthesized by transaldolase when ribose 5-phosphate and fructose 6-phosphate were used as acceptor and donor substrates, respectively (Creek *et al.*, 2012; Creek *et al.*, 2015). Using genetic approaches, the study with yeast by Clasquin *et al.* (2011) suggested that D-*glycero*-D-*ido*-octulose 1,8-bisphosphate might be synthesized by the aldol addition of dihydroxyacetone phosphate (DHAP) and ribose 5-phosphate, which is catalysed by the ubiquitous glycolytic enzyme fructose bisphosphate aldolase (Clasquin *et al.*, 2011). This is consistent with the modified Calvin–Benson–Bassham pathways proposed by Flanigan *et al.* (2006) and Williams and MacLeod (2006).

**Box 1. *In vitro* synthesis of octulose**

D-*glycero*-D-*altro*-octulose, L-*glycero*-L-*galacto*-octulose and D-*glycero*-L-*gluco*-octulose have been synthesized in a condensation reaction catalysed by rabbit muscle aldolase using dihydroxyacetone phosphate (DHAP) with D-ribose, L-arabinose and D-lyxose, respectively (Jones and Sephton, 1960). Similarly, D-*glycero*-D-*manno*-octulose has been synthesized in a reaction using D-ribose, D-fructose 1,6-bisphosphate and rabbit muscle aldolase (Haustveit, 1976). In addition, Paoletti *et al.* (1979) used rabbit muscle aldolase to synthesize D-*glycero*-D-*altro*-octulose 1,8-bisphosphate or D-*glycero*-D-*ido*-octulose 1,8-bisphosphate by condensation of DHAP with ribose 5-phosphate or arabinose 5-phosphate. While studying methylthiolincosamide biosynthesis, Sasaki *et al.* (2012) observed that D-*glycero*-D-*altro*-octulose is formed via a transaldol reaction catalysed by a putative transaldolase (LmbR protein from *Streptomyces lincolnensis*) using D-fructose 6-phosphate or D-sedoheptulose 7-phosphate as the C3 donor and D-ribose 5-phosphate as the C5 acceptor.

**Figure:**
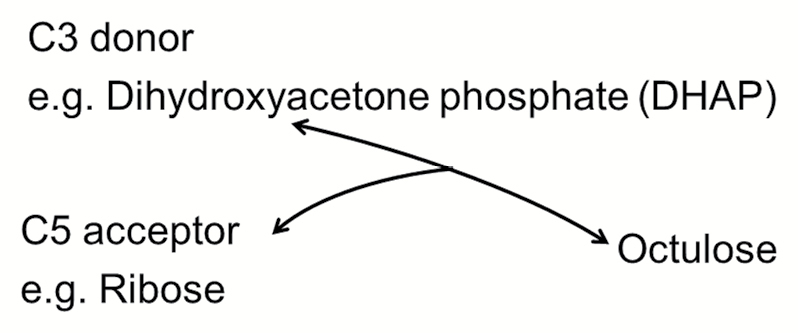

Various octulose isomers can be found in a single plant species (Table 1). Octulose synthesis can be catalysed by different enzymes, including aldolase, transaldolase or transketolase. However, it is also possible that different octulose isomers are synthesized in a similar way only with substrates of specific stereo configurations, as with the synthesis of D-*glycero*-D-*manno*-octulose and D-*glycero*-L-*galacto*-octulose in avocado and *Sedum* species (Charlson and Richtmyer, 1960; Sephton and Richtmyer, 1963*a*). Whether aldolase, transaldolase or transketolase catalyses the synthesis of octulose could be verified by transgenic approaches. Similar studies have been done in potato and tobacco (Haake *et al.* 1998; Henkes *et al.* 2001). The genetic transformation of octulose-containing plants, such as *Spinacia oleracea* or *Medicago sativa*, is possible and thus aldolase or transketolase mutants could be generated for analyzing octulose metabolism.

## The missing loop: kinases and phosphatases

Many monosaccharides are recognized via their phosphate-ester derivatives, which are important intermediates in the catalysis and synthesis of carbohydrates in living organisms (Robyt, 1998). To participate in various aspects of metabolism, free monosaccharides are activated by an initial phosphorylation which is catalysed by sugar kinases. For example, hexokinase catalyses the phosphorylation of glucose to form glucose 6-phosphate and plays a central role in glucose sensing (Rolland *et al.*, 2006). It is reasonable to hypothesize that there are also kinases catalysing the phosphorylation of octulose. In the resurrection plant *C. plantagineum*, the most abundant sugar in hydrated plants, D-*glycero*-D-*ido*-octulose, is converted to sucrose during dehydration and the phosphorylation of octulose should be essential in this process. However, the enzymatic activity of an octulose kinase has not yet been reported. As mentioned in discussion of its synthesis, octulose is involved in both photosynthesis and pentose phosphate pathways. Therefore, it is reasonable to hypothesize one or more phosphatases that dephosphorylate mono- and bisphosphates of octulose to produce free octulose, especially in plants which accumulate octulose. For some free monosaccharides, specific phosphatases exist to catalyse the dephosphorylation of their phosphate-ester derivatives (e.g. glucose, galactose and sedoheptulose: Van Schaftingen and Gerin, 2002; Conklin *et al.*, 2006; Ceusters *et al.*, 2013). To date, various sugar phosphatases have been identified, including L-galactose 1-phosphate phosphatase in Arabidopsis (Conklin *et al.*, 2006). However, the phosphatases involved in octulose production have not been explored.

Besides traditional protein purification, which provides primary solutions for new protein characterization (Janson, 2012), knowledge from genomics, transcriptomics, proteomics and metabolomics, and approaches from genetic modification, have enhanced the identification and characterization of specific sugar kinases and phosphatases. For example, Groisillier *et al.* (2014) revealed that haloalkanoic dehalogenase-like enzymes in brown algae have specific mannitol-1-phosphatase activity, and Conklin *et al.* (2006) reported that the Arabidopsis *VTC4* gene, which has been annotated as encoding a myo-inositol monophosphatase-like protein, encodes L-galactose 1-phosphate phosphatase. Similar approaches are possible for octulose-containing plant species, especially *Spinacia oleracea and Medicago sativa*, where there is full genome information and they can easily be genetically modified. Studies of the kinases and phosphatases involved in octulose metabolism might provide novel insights into carbohydrate metabolism in the same way that studies on sedoheptulose, a seven-carbon monosaccharide with a similar structure as octulose, have expanded basic understanding of carbohydrate metabolism (Clasquin *et al.*, 2011; Nagy and Haschemi, 2013).

## Interconnections between octulose and other monosaccharides

In the complex metabolic network of plants, the metabolism of octulose is probably interconnected with other metabolites, particularly seven- and nine-carbon sugars. When Begbie and Richtmyer (1966) and Sephton and Richtmyer (1963*a*) first isolated octulose in avocado and *Primula officinalis*, they also found heptoses, heptuloses and nonuloses. Numerous studies have shown how variations among family members of biosynthetic enzymes result in substrate preferences and further lead to the diversity of phytochemicals in a given plant species (Pichersky and Gang, 2000).

Octulose synthesis is catalysed by transketolase, transaldolase or fructose bisphosphate aldolase *in vitro*. All these enzymes can accept various substrates (Krüger and von Schaewen, 2003; Samland and Sprenger, 2009). Our study showed that the recombinant *C. plantagineum* transketolase 7 and 10 can catalyse the formation of both octulose phosphate and sedoheptulose phosphate (Zhang *et al.*, 2016). Being important components of the Calvin cycle and the pentose phosphate pathway, reactions involving sedoheptulose are of primary endosymbiotic origin in plastids of eukaryotes (Richards *et al.*, 2006; Price *et al.*, 2012). Therefore it can be speculated that the metabolism of eight-carbon sugars might be derived from seven-carbon sugars. The study of Clasquin *et al.* (2011) provides a genetic proof for this hypothesis. They showed that in yeast the sedoheptulose-1,7-bisphosphatase deletion mutant accumulates sedoheptulose-1,7-bisphosphate and octulose-1,8-bisphosphate; the enzyme is both a selective sedoheptulose bisphosphatase and a selective octulose bisphosphatase. Another example of an interconnection between seven- and eight-carbon sugars is the presence of 3-deoxy-D-arabino-heptulosonic acid (DAH) and 3-deoxy-D-manno-octulosonic acid (KDO). DAH 7-phosphate is synthesized by an aldol condensation of phosphoenolpyruvate (PEP) and erythrose 4-phosphate catalysed by DAH 7-phosphate synthase, while KDO 8-phosphate is synthesized by an aldol condensation of PEP and arabinose 5-phosphate catalysed by KDO 8-phosphate synthase. Both reactions appear to occur by a common mechanism involving an attack from the ‘si’ face of PEP onto the ‘re’ face of the aldehyde group of the monosaccharide, followed by an attack by water onto the C2 position of PEP (Furdui *et al.*, 2005). Based on sequence similarity and X-ray crystal structures, Birck and Woodard (2001) proposed that KDO 8-phosphate synthase and DAH 7-phosphate synthase are the result of a divergent evolutionary process from a common ancestor. These studies provide new evidence that the metabolism of seven-carbon sugars might be derived from eight-carbon sugars or vice versa. Thus studies on octulose, such as identification of specific phosphatases, could borrow ideas from those of sedoheptulose.

## Physiological functions

Although studies on octulose have not received much attention, the sugar may be universal in the three main life-forms (microbes, plants and animals). As well as in plants, as already discussed, it has been found in bacteria (e.g. *Streptomyces lincolnensis*; Sasaki *et al.*, 2012), fungi (e.g. *Saccharomyces cerevisiae*; Clasquin *et al.*, 2011), a protozoan parasite (*Trypanosoma brucei*; Creek *et al.*, 2012), animals (rat liver; Paoletti *et al.*, 1979), human red blood cells (Bartlett and Bucolo, 1960) and human erythrocytes (Vanderheiden, 1965). Therefore, it is reasonable to assume that octulose is in fact much more widespread among living organisms.

As already noted, about forty years ago Williams *et al.* (1978) proposed an L-type pentose phosphate pathway involving octulose phosphates in liver cells. Although, only limited confirmation of this pathway has been obtained, the discoveries of sedoheptulokinase (Wamelink *et al.*, 2008) and sedoheptulose 1,7-bisphosphatase (Clasquin *et al.*, 2011) demonstrate that the full biochemical spectrum of the pentose phosphate pathway exceeds what we currently know (Stincone *et al.*, 2015). Octulose could play an important role in the extended pentose phosphate pathway, and this needs further investigation.

Abundant octulose may also function as carbohydrate storage similar to starch (Norwood *et al.*, 2000). Our studies showed that 75–80% of the sugars in phloem exudate of *C. plantagineum* is D-*glycero*-D-*ido*-octulose, indicating that this might be an important sugar transport form in this species (Zhang *et al.*, 2016). It is intriguing that the desiccation-tolerant species in the genus *Lindernia* (e.g. *L. brevidens*) have significantly higher levels of D-*glycero*-D-*ido*-octulose than desiccation-sensitive species (e.g. *L. subracemosa* and *L. rotundifolia*): the presence of octulose correlates with desiccation tolerance. A reason for its high accumulation in some species could be that D-*glycero*-D-*ido*-octulose has a hydroxyl-scavenging ability superior to other common sugars (e.g. sucrose) (Zhang and Bartels, 2016). Such speculation needs to be proven directly. Although tools for direct genetic modifications are unsuited or lacking for resurrection plants, progress might be possible using specific inhibitors to limit octulose production and through this confirming its physiological role. For instance, oxythiamine can act as an inhibitor of transketolase, a key enzyme for octulose synthesis (Wang *et al.*, 2013). Similar to the experiments done with sucrose by Matros *et al.* (2015), analogues of octulose can also be used in hydroxyl-scavenging reactions in plants *in vivo* to assess the function of octulose as an antioxidant.

## Commercial value

Could octulose have beneficial effects in nutrition and healthcare for animals or people? One possibility is as an antioxidant: a derivative of octulose, 3,7-anhydro-1-deoxy-D-*glycero*-D-*gulo*-2-octulose, which was isolated from the roots of *Brassica rapa*, showed significant ROS reduction and protective effects on glutamate-induced cell death in neuronal HT-22 nerve cells (Wu *et al.*, 2013). It may also be valuable in direct disease resistance: as proposed by Kovářová and Barrett (2016), some enzymes of the pentose phosphate pathway (possibly including enzymes linked to octulose biosynthesis) are essential to the parasites *Trypanosoma brucei*, *Trypanosoma cruzi* and various *Leishmania* species, and offer potential targets for new drugs (inhibitors) against one or other of the diseases caused by these parasites. It is also worth exploring the role of octulose in carbohydrate metabolism in higher animals and humans, as it is often ingested from different foods (e.g. alfalfa plants and avocado fruits). Last, more research on associated metabolic pathways and biological activities (e.g. antibacterial, antifungal, antiviral and anticancer) of octulose and its derivatives might be beneficial for developing novel drugs and cosmetics.
